# Supplementary material for: The online delivery of exercise oncology classes supported with health coaching: a parallel pilot randomized controlled trial
Source: Pilot Feasibility Stud. 2023 May 12;9:82. doi: 10.1186/s40814-023-01316-z (PMC10175911; doi:10.1186/s40814-023-01316-z)
Supplement: Supplementary file 2 — Additional file 2. Health Coaching Protocol. [file 40814_2023_1316_MOESM2_ESM.docx]

S2: Health Coaching Protocol

**Title:** The online delivery of exercise oncology classes supported with health coaching: A pilot randomized controlled trial.

**Journal:** Journal of Behavioural Medicine

**Authors:** Maximilian Eisele^1^, Rosie Twomey^1,2^, Andrew J. Pohl^1^, Meghan H. McDonough^1^, Margaret L. McNeely^3^, Manuel Ester^1^, Julia T. Daun^1^, S. Nicole Culos-Reed^1,4,5^

**Author Affiliations:**

1 Faculty of Kinesiology, University of Calgary, AB, Canada

2 Cumming School of Medicine, University of Calgary, AB, Canada

3 Department of Physical Therapy & Oncology, University of Alberta, AB, Canada

4 Department of Oncology, Cummings School of Medicine, University of Calgary, AB, Canada

5 Department of Psychosocial Resources, Tom Baker Cancer Centre, Cancer Care, Alberta Health Services, AB, Canada

**Corresponding Author:** Maximilian Eisele ([maximilian.eisele@ucalgary.ca](mailto:maximilian.eisele@ucalgary.ca))

Health Coaching:

Participants randomized to the HC intervention received weekly individual HC calls. HC was structured based on Wolever et al. (2013) definition, which emphasizes that HC has to be participant-centred, built on a coach-participant relationship, and include participant-determined goals, a self-discovery process to find solutions, patient accountability, and education. A day before each HC call, the participants received a short survey (Survey Monkey platform, Momentive Inc.; San Mateo, CA) on fatigue, QoL, stress, loneliness, and social support, enabling tailoring of the HC call to the individual.

Educational topics within the HC calls included: Goal Setting, Monitoring Behaviour, Barrier Management, Social Support, Stress Management, Adapting the Program, Self-compassion, Sleep & Nutrition, Reflection, Health Media, Remote Resources, and Maintaining Motivation. The order of the educational topics could be adjusted based on the specific participants’ needs each week. Based on the interests of the HC calls in the 8-week wave, the educational topics Self-Compassion, Sleep & Nutrition, Reflection, and Health Media were added to the 12-week wave. Each HC call was structured, starting off with a reflection on the previous week, a conversation about the educational topic, and finishing with an action plan for the upcoming week. Based on participant interest, a summary sheet of the educational topic was sent to the individual. At the half-way point of the intervention, the participant provided feedback on the HC calls, ensuring optimization of HC. The health coaches were graduate students trained in behaviour change strategies, exercise oncology, had extensive experience with the larger ACE program, and completed at least 30 hours of HC specific training (including mock interviews, motivational interviewing materials, and a literature review). The weekly HC calls were held via Zoom and at a convenient time for the participant. The length of each call was dependent on the participant’s needs. No restrictions on receiving additional counselling or coaching from outside sources were made.

Table 1. Behaviour change techniques (BCT) incorporated in the online maintenance program

| BCT domain | BCTs |
| --- | --- |
| 1. Goal setting and planning |  |
|  | 1.1 Goal setting (behaviour) |
|  | 1.2 Problem solving |
|  | 1.3 Goal setting (outcome) |
|  | 1.4 Action planning |
|  | 1.5 Review behaviour goals |
|  | 1.6 Discrepancy between current behaviour and goal |
|  | 1.7 Review outcome goals |
|  | 1.9 Commitment |
| 2. Feedback and monitoring |  |
|  | 2.2 Feedback on behaviour |
|  | 2.3 Self-Monitoring of behaviour |
|  | 2.4 Self-monitoring of outcome of behaviour |
|  | 2.7 Feedback on outcome of behaviour |
| 3. Social Support |  |
|  | 3.1 Social support (unspecified) |
|  | 3.2 Social support (practical) |
|  | 3.3 Social support (emotional) |
| 4. Shaping knowledge |  |
|  | 4.1 Instruction on how to perform a behaviour |
| 5. Natural consequences |  |
|  | 5.1 Information about health consequences |
|  | 5.4 Monitoring of emotional consequences |
|  | 5.6 Information about emotional consequences |
| 6. Comparison of behaviour |  |
|  | 6.1 Demonstration of the behaviour |
| 8. Repetition and substitution |  |
|  | 8.2 Behavior substitution |
|  | 8.6 Generalization of a target behaviour |
|  | 8.7 Graded tasks |
| 9. Comparison of outcomes |  |
|  | 9.1 Credible source |
| 10. Reward and threat |  |
|  | 10.4 Social reward |
| 13. Identity |  |
|  | 13.2 Framing/ reframing |
| 15. Self-belief |  |
|  | 15.1 Verbal persuasion about capability |
|  | 15.3 Focus on past successes |

*BCTs are based on Michie et al. (2013) Behaviour Change Taxonomy

Health Coaching Fidelity Check List

**1^st^ WEEK**

Covered:

1. Chat about current situation (Time spent: ____________)
   - Listen
   - Participant focus

*Comment:*

1. Process evaluation (Time spent: ____________)
   - How are you doing? Energy?

- How much PA last week?
- How did the exercises go?
- Any barriers
- Highlight or favourite exercise of last week

*Comment:*

1. Set / Update Goals (self-determined) (Time spent: ____________)
   - Led by participant whatever they think is achievable
     - Tailor to individual (before) 🡪 use as options in the end led by participant
   - Motivate
   - Short education on **Goal Setting**

- Importance: provides guidance and mindfulness
- Setting SMART^2^ goals
  - **Specific:** What exactly do you want to accomplish? Where? When? Why? How much?
  - **Measurable:** Are you able to assess your progress?
  - **Attainable:** Is your goal within your reach given your current situation?
  - **Realistic:** Are you both willing and able to work towards your goal?
  - **Timely**: What is the deadline for completing your goal?
  - **Together**: Who is supporting you to be active?
- Important to consider:
  - Set long-term and short-term goals
  - Challenge yourself
  - Focus on the process
  - Re-evaluate your progress and goals frequently
- Participant questions:
  - Do you have any goals already?
  - If not, think of your goals over the next week
- If participant would like to have more information: pg. 8

*Comment:*

1. Accountability plan (Time spent: ____________)
   - Establish potential barriers
   - Discuss options of overcoming these
     - List of potential actions
   - Develop a strategy plan

*Comment:*

1. Summarize Goals & Overcoming barrier strategies (Time spent: ____________)
   - Positive outlook

*Comment:*

**2^nd^ WEEK**

Covered:

1. Chat about current situation (Time spent: ____________)
   - Listen
   - Participant focus

*Comment:*

1. Process evaluation (Time spent: ____________)
   - How are you doing? Energy?

- How much PA last week?
- How did the exercises go?
- Any barriers
- Highlight or favourite exercise of last week

*Comment:*

1. Set / Update Goals (self-determined) (Time spent: ____________)
   - Led by participant whatever they think is achievable
     - Tailor to individual (before) 🡪 use as options in the end led by participant
   - Motivate

- Short education on **Monitoring Behaviour**
- Importance: Allows for instant feedback on previously set goals, elevates awareness
- Options:
  - Exercise Log/Diary
  - Smart watch
  - Tracking App
  - Simply taking some time at night to think about accomplishments
- Important to consider:
  - Don’t evaluate only observe
  - Make it a continuous habit
- Participant questions:
  - Are you using anything already to track PA levels?
    - How often?
  - Which method of tracking would you prefer?
- If participant would like to have more information: pg. 11

*Comment:*

1. Accountability plan (Time spent: ____________)
   - Establish potential barriers
   - Discuss options of overcoming these
     - List of potential actions
   - Develop a strategy plan

*Comment:*

1. Summarize Goals & Overcoming barrier strategies (Time spent: ____________)
   - Positive outlook

*Comment:*

**3^rd^ WEEK**

Covered:

1. Chat about current situation (Time spent: ____________)
   - Listen
   - Participant focus

*Comment:*

1. Process evaluation (Time spent: ____________)
   - How are you doing? Energy?

- How much PA last week?
- How did the exercises go?
- Any barriers
- Highlight or favourite exercise of last week

*Comment:*

1. Set / Update Goals (self-determined) (Time spent: ____________)
   - Led by participant whatever they think is achievable
     - Tailor to individual (before) 🡪 use as options in the end led by participant
   - Motivate

- Short education on **Barrier Management**
- Importance: Planning for barriers may help you cope with them better
- 9 most common Barriers in CS:
  - Lack of Self discipline
  - Fatigue
  - Lack of Time
  - Exercise not a Priority
  - Lack of enjoyment
  - Lack of interest
  - Lack of encouragement
  - Bad weather
  - Tired
- Make dependent on individual’s barriers
- Participant Questions:
  - Which barriers do you anticipate?
  - What is the most common reason for you not to exercise?
- If participant would like to have more information: pg. 13

*Comment:*

1. Accountability plan (Time spent: ____________)
   - Establish potential barriers
   - Discuss options of overcoming these
     - List of potential actions
   - Develop a strategy plan

*Comment:*

1. Summarize Goals & Overcoming barrier strategies (Time spent: ____________)
   - Positive outlook

*Comment:*

**4^th^ WEEK**

Covered:

1. Chat about current situation (Time spent: ____________)
   - Listen
   - Participant focus

*Comment:*

1. Process evaluation (Time spent: ____________)
   - How are you doing? Energy?

- How much PA last week?
- How did the exercises go?
- Any barriers
- Highlight or favourite exercise of last week

*Comment:*

1. Set / Update Goals (self-determined) (Time spent: ____________)
   - Led by participant whatever they think is achievable
     - Tailor to individual (before) 🡪 use as options in the end led by participant
   - Motivate

- Short education on **Social Support**
- Importance: Is a crucial piece in supporting long lasting behaviour change
- Can be in many forms:
  - Family members
  - Friends
  - Even Dogs
- Be participant specific ask about their loved ones (with names) let them guide the conversation
  - Does … support you to stay active
  - How? Are they exercising with you?
  - What would you like to change?
- If participant would like to have more information: pg. 17

*Comment:*

1. Accountability plan (Time spent: ____________)
   - Establish potential barriers
   - Discuss options of overcoming these
     - List of potential actions
   - Develop a strategy plan

*Comment:*

1. Summarize Goals & Overcoming barrier strategies (Time spent: ____________)
   - Positive outlook

*Comment:*

**5^th^ WEEK**

Covered:

1. Chat about current situation (Time spent: ____________)
   - Listen
   - Participant focus

*Comment:*

1. Process evaluation (Time spent: ____________)
   - How are you doing? Energy?

- How much PA last week?
- How did the exercises go?
- Any barriers
- Highlight or favourite exercise of last week

*Comment:*

1. Set / Update Goals (self-determined) (Time spent: ____________)
   - Led by participant whatever they think is achievable
     - Tailor to individual (before) 🡪 use as options in the end led by participant
   - Motivate

- Short education on **Stress Management**
- Importance: Coping mechanisms
- Eustress vs Distress
- Relaxation training
  - Structured process that requires practice
- Examples:
  - Body scan
  - Progressive Muscle relaxation
  - Meditation
  - Yoga
  - Stretching
  - Self-hypnosis
  - Prayer
  - Music / Sound
  - Diaphragmatic breathing
- Mind Body Connection
- Participant question:
  - Have you tried these techniques?
  - Awareness of Body/Mind state?
- If participant would like to have more information: pg. 22

*Comment:*

1. Accountability plan (Time spent: ____________)
   - Establish potential barriers
   - Discuss options of overcoming these
     - List of potential actions
   - Develop a strategy plan

*Comment:*

1. Summarize Goals & Overcoming barrier strategies (Time spent: ____________)
   - Positive outlook

*Comment:*

**6^th^ WEEK**

Covered:

1. Chat about current situation (Time spent: ____________)
   - Listen
   - Participant focus

*Comment:*

1. Process evaluation (Time spent: ____________)
   - How are you doing? Energy?

- How much PA last week?
- How did the exercises go?
- Any barriers
- Highlight or favourite exercise of last week

*Comment:*

1. Set / Update Goals (self-determined) (Time spent: ____________)
   - Led by participant whatever they think is achievable
     - Tailor to individual (before) 🡪 use as options in the end led by participant
   - Motivate

- Short education on **Adapting the Program**
- Importance: Variation makes exercise more exciting and providing different stimuli for your muscles is actually beneficial
- Possible opportunities for adapting your program (FITT- Principle)
  - **FREQUENCY**

Add an extra day of exercise to your week. Add a few more repetitions, or even another set to your resistance exercises.

- - **INTENSITY**

Work a little harder than you have been. Increase the weight of your resistance exercises or add some jogging into your daily walks. Continue to challenge yourself but stay within the recommended intensity guidelines.

- - **TYPE**

Switch around your exercises – try different exercises, move from resistance bands to dumbbells or start cycling instead of walking. Try a yoga or indoor cycling class. Variety!

- - **TIME**

Increase the total amount of time of your average exercise session.

- Participant Question:
  - Have you already tried adapting the home program?
- Potentially explain how varying the home program works
  - Exercise Appendix
  - Recommended options behind each Exercise
- If participant would like to have more information: pg. 15

*Comment:*

1. Accountability plan (Time spent: ____________)
   - Establish potential barriers
   - Discuss options of overcoming these
     - List of potential actions
   - Develop a strategy plan

*Comment:*

1. Summarize Goals & Overcoming barrier strategies (Time spent: ____________)
   - Positive outlook

*Comment:*

**7^th^ WEEK**

Covered:

1. Chat about current situation (Time spent: ____________)
   - Listen
   - Participant focus

*Comment:*

1. Process evaluation (Time spent: ____________)
   - How are you doing? Energy?

- How much PA last week?
- How did the exercises go?
- Any barriers
- Highlight or favourite exercise of last week

*Comment:*

1. Set / Update Goals (self-determined) (Time spent: ____________)
   - Led by participant whatever they think is achievable
     - Tailor to individual (before) 🡪 use as options in the end led by participant
   - Motivate
   - Short education on **Self-Compassion**

- Importance: Key to treating yourself healthy
- Giving same kindness to ourselves that we would give to others
- 3 Elements:
  - Self-kindness vs Self-judgment
  - Common Humanity vs. Isolation
  - Mindfulness vs Over-identification
- Strategies:
  - Breathing
  - Petting a pet
  - Cup of tea
- Participant question:
  - Friend is struggling. How would you respond?
  - You’re struggling. How do you respond to yourself?
  - Differences Similarities?

*Comment:*

1. Accountability plan (Time spent: ____________)
   - Establish potential barriers
   - Discuss options of overcoming these
     - List of potential actions
   - Develop a strategy plan

*Comment:*

1. Summarize Goals & Overcoming barrier strategies (Time spent: ____________)
   - Positive outlook

*Comment:*

**8^th^ WEEK (A)**

Covered:

1. Chat about current situation (Time spent: ____________)
   - Listen
   - Participant focus

*Comment:*

1. Process evaluation (Time spent: ____________)
   - How are you doing? Energy?

- How much PA last week?
- How did the exercises go?
- Any barriers
- Highlight or favourite exercise of last week

*Comment:*

1. Set / Update Goals (self-determined) (Time spent: ____________)
   - Led by participant whatever they think is achievable
     - Tailor to individual (before) 🡪 use as options in the end led by participant
   - Motivate
   - Short education on **Sleep**

- Importance: Sleep helps us recover and rebuild physically and mentally
- Focus:
  - Routine
  - Length
  - Conditions
  - Strategies
- Participant questions:
  - What is your daily routine?
  - What do you do before you go to bed?
  - What would be your ideal length? When do you feel most rested?

*Comment:*

1. Accountability plan (Time spent: ____________)
   - Establish potential barriers
   - Discuss options of overcoming these
     - List of potential actions
   - Develop a strategy plan

*Comment:*

1. Summarize Goals & Overcoming barrier strategies (Time spent: ____________)
   - Positive outlook

*Comment:*

**8^th^ WEEK (B)**

Covered:

1. Chat about current situation (Time spent: ____________)
   - Listen
   - Participant focus

*Comment:*

1. Process evaluation (Time spent: ____________)
   - How are you doing? Energy?

- How much PA last week?
- How did the exercises go?
- Any barriers
- Highlight or favourite exercise of last week

*Comment:*

1. Set / Update Goals (self-determined) (Time spent: ____________)
   - Led by participant whatever they think is achievable
     - Tailor to individual (before) 🡪 use as options in the end led by participant
   - Motivate
   - Shor education on **Nutrition**

- Importance: Can affect weight, strength, and energy
- Focus:
  - Balanced meals
  - Fresh
  - Calories
  - Caution with supplements
  - Drinking enough
- Participant questions:
  - What are your eating habits? Time? Place? Together?
  - What are things you would want to change and why?
  - Strategies? Meal prepping? Grocery list? Big water bottle?

*Comment:*

1. Accountability plan (Time spent: ____________)
   - Establish potential barriers
   - Discuss options of overcoming these
     - List of potential actions
   - Develop a strategy plan

*Comment:*

1. Summarize Goals & Overcoming barrier strategies (Time spent: ____________)
   - Positive outlook

*Comment:*

**9^th^ WEEK**

Covered:

1. Chat about current situation (Time spent: ____________)
   - Listen
   - Participant focus

*Comment:*

1. Process evaluation (Time spent: ____________)
   - How are you doing? Energy?

- How much PA last week?
- How did the exercises go?
- Any barriers
- Highlight or favourite exercise of last week

*Comment:*

1. Set / Update Goals (self-determined) (Time spent: ____________)
   - Led by participant whatever they think is achievable
     - Tailor to individual (before) 🡪 use as options in the end led by participant
   - Motivate

- Short education on **Reflection**
- Importance: We often want to strive for more but seldom take the time to celebrate how far we’ve come
- Focus:
  - Gibb’s Reflection cycle
  - Learning from past successes and failures
  - Readjusting your goals
- Participant questions:
  - What are you proud of?
  - Where do you see yourself on the journey to your previously established goals?
  - What are ways in which you can readjust or build upon your goals?

*Comment:*

1. Accountability plan (Time spent: ____________)
   - Establish potential barriers
   - Discuss options of overcoming these
     - List of potential actions
   - Develop a strategy plan

*Comment:*

1. Summarize Goals & Overcoming barrier strategies (Time spent: ____________)
   - Positive outlook

*Comment:*

**10^th^ WEEK**

Covered:

1. Chat about current situation (Time spent: ____________)
   - Listen
   - Participant focus

*Comment:*

1. Process evaluation (Time spent: ____________)
   - How are you doing? Energy?

- How much PA last week?
- How did the exercises go?
- Any barriers
- Highlight or favourite exercise of last week

*Comment:*

1. Set / Update Goals (self-determined) (Time spent: ____________)
   - Led by participant whatever they think is achievable
     - Tailor to individual (before) 🡪 use as options in the end led by participant
   - Motivate

- Short education on **Health Media**
- Importance: There is a lot of information online but a lot of it is also wrong or unsafe for you 🡪 creating awareness of which information is reliable
- Consider:
  - Reliable sources:
    - Government designation or degree
      - Certified Exercise Physiologist (CEP)
      - Registered Dietitian (RD)
    - Does the person claiming something use peer-reviewed references?
  - Questionable sources:
    - Nutritionist
      - Don’t need certificate or even degree
    - Any radical breakthrough results
    - Social media adds
  - Are the sources cancer specific?
- Don’t hesitate to ask any of our CEPs about information you found online
- If participant would like to have more information: pg. 16

*Comment:*

1. Accountability plan (Time spent: ____________)
   - Establish potential barriers
   - Discuss options of overcoming these
     - List of potential actions
   - Develop a strategy plan

*Comment:*

1. Summarize Goals & Overcoming barrier strategies (Time spent: ____________)
   - Positive outlook

*Comment:*

**11^th^ WEEK**

Covered:

1. Chat about current situation (Time spent: ____________)
   - Listen
   - Participant focus

*Comment:*

1. Process evaluation (Time spent: ____________)
   - How are you doing? Energy?

- How much PA last week?
- How did the exercises go?
- Any barriers
- Highlight or favourite exercise of last week

*Comment:*

1. Set / Update Goals (self-determined) (Time spent: ____________)
   - Led by participant whatever they think is achievable
     - Tailor to individual (before) 🡪 use as options in the end led by participant
   - Motivate

- Short education on **Remote Resources**
- ACE maintenance package with video future
- Wellspring offers remote programs
  - @  <https://wel.gametime.net/auth>
- Any other cancer specific remote resources?
- Ask participant questions about experience with these
  - Did you try any of them?
  - What were your thoughts?
- If participant would like to have more information: pg. 18

*Comment:*

1. Accountability plan (Time spent: ____________)
   - Establish potential barriers
   - Discuss options of overcoming these
     - List of potential actions
   - Develop a strategy plan

*Comment:*

1. Summarize Goals & Overcoming barrier strategies (Time spent: ____________)
   - Positive outlook

*Comment:*

**12^th^ WEEK**

Covered:

1. Chat about current situation (Time spent: ____________)
   - Listen
   - Participant focus

*Comment:*

1. Process evaluation (Time spent: ____________)
   - How are you doing? Energy?

- How much PA last week?
- How did the exercises go?
- Any barriers
- Highlight or favourite exercise of last week

*Comment:*

1. Set / Update Goals (self-determined) (Time spent: ____________)
   - Led by participant whatever they think is achievable
     - Tailor to individual (before) 🡪 use as options in the end led by participant
   - Motivate

- Short education on **Maintaining Motivation & Remote Resources**
- Importance: key tool required to achieve lifelong fitness
- Key sources of motivation
  - Fun, enjoyment, stimulation
  - A feeling of accomplishment (Meaningfulness)
  - The pleasure of learning
  - A well-identified benefit such as sleeping better and feeling calmer
- Aim to fuel these sources by
  - Being mindful
    - While Exercising
    - While making healthy life choices
  - Setting Goals/ Making it meaningful
  - Monitoring your process
- Participant question:
  - What motivates you to keep exercising?
- If participant would like to have more information: pg. 19

*Comment:*

1. Accountability plan (Time spent: ____________)
   - Establish potential barriers
   - Discuss options of overcoming these
     - List of potential actions
   - Develop a strategy plan

*Comment:*

1. Summarize Goals & Overcoming barrier strategies (Time spent: ____________)
   - Positive outlook

*Comment:*

Michie, S., Richardson, M., Johnston, M., Abraham, C., Francis, J., Hardeman, W., Eccles, M. P., Cane, J., & Wood, C. E. (2013). The behavior change technique taxonomy (v1) of 93 hierarchically clustered techniques: building an international consensus for the reporting of behavior change interventions. *Annals of Behavioral Medicine, 46*(1), 81-95.

Wolever, R. Q., Simmons, L. A., Sforzo, G. A., Dill, D., Kaye, M., Bechard, E. M., Southard, M. E., Kennedy, M., Vosloo, J., & Yang, N. (2013). A Systematic Review of the Literature on Health and Wellness Coaching: Defining a Key Behavioral Intervention in Healthcare. *Global advances in health and medicine, 2*(4), 38-57. <https://doi.org/10.7453/gahmj.2013.042>
