# Supplementary material for: The online delivery of exercise oncology classes supported with health coaching: a parallel pilot randomized controlled trial
Source: Pilot Feasibility Stud. 2023 May 12;9:82. doi: 10.1186/s40814-023-01316-z (PMC10175911; doi:10.1186/s40814-023-01316-z)
Supplement: Supplementary file 3 — Additional file 3. Garmin Wear Time Graph. [file 40814_2023_1316_MOESM3_ESM.docx]

S3: Garmin Wear Time Graph

**Title:** The online delivery of exercise oncology classes supported with health coaching: A pilot randomized controlled trial.

**Journal:** Journal of Behavioural Medicine

**Authors:** Maximilian Eisele^1^, Rosie Twomey^1,2^, Andrew J. Pohl^1^, Meghan H. McDonough^1^, Margaret L. McNeely^3^, Manuel Ester^1^, Julia T. Daun^1^, S. Nicole Culos-Reed^1,4,5^

**Author Affiliations:**

1 Faculty of Kinesiology, University of Calgary, AB, Canada

2 Cumming School of Medicine, University of Calgary, AB, Canada

3 Department of Physical Therapy & Oncology, University of Alberta, AB, Canada

4 Department of Oncology, Cummings School of Medicine, University of Calgary, AB, Canada

5 Department of Psychosocial Resources, Tom Baker Cancer Centre, Cancer Care, Alberta Health Services, AB, Canada

**Corresponding Author:** Maximilian Eisele ([maximilian.eisele@ucalgary.ca](mailto:maximilian.eisele@ucalgary.ca))

Figure 1. Percent Wear time per Week in the 8-Week and the 12-Week Wave
