# Supplementary material for: The online delivery of exercise oncology classes supported with health coaching: a parallel pilot randomized controlled trial
Source: Pilot Feasibility Stud. 2023 May 12;9:82. doi: 10.1186/s40814-023-01316-z (PMC10175911; doi:10.1186/s40814-023-01316-z)
Supplement: Supplementary file 5 — Additional file 5. OSF1: Exploratory Outcome Measures and Analysis. [file 40814_2023_1316_MOESM5_ESM.pdf]

OSF1: Exploratory Outcome Measures and Analysis

EXPLORATORY OUTCOME MEASURES

Moderate to Vigorous Physical Activity Minutes

MVPA was assessed objectively and through self-report. The modified Godin Leisure Time Exercise Questionnaire (mGLTEQ; Amireault et al., 2015) was used to assess self-reported MVPA per week at pre and post intervention. The Garmin Vivosmart4 was used as an objective measure of weekly MVPA throughout the study based on Garmin's proprietary algorithm. To be considered for weekly objective MVPA calculation, the activity tracker had to have been worn for at least four valid days. A valid day was judged by wearing the tracker for at least 10h a day (Gell et al., 2017; Hartman et al., 2018) with non-wear time being defined as 60 consecutive minutes (Matthews et al., 2008). If the tracker was worn more than four days but not the complete seven days, the days of non-wear time were imputed by taking the average of the valid recorded days within that week. Adherence to exercise guidelines was determined by comparing the weekly objective minutes of MVPA to the oncology exercise guidelines of 90-minutes of MVPA per week (Campbell et al., 2019).

Physical functioning

Physical functioning assessments were administered via Zoom before (week 0) and after the intervention (week 9 or week 13 in the two waves, respectively) by a CEP blind to intervention allocation. The ACE testing protocol (McNeely et al., 2020) was modified to ensure safety during the online assessment and account for limitations in equipment in a home setting. Specifically, the cardiovascular fitness assessment was a 2-minute step test (instead of the 6-minute walk test used in ACE), the lower extremity flexibility assessment was a chair sit and reach test (instead of the box sit and reach test), and height and weight were self-reported (instead of measured by the research staff). Tests removed from the in-person protocol included grip strength and waist and hip circumference. Measures that followed the same procedures as during the in-person assessment included upper extremity flexibility (shoulder range of motion), balance (a one-legged stance with open eyes), and muscular endurance (30 second sit-to-stand; McNeely et al., 2020).

Upper extremity flexibility

Shoulder range of motion (ROM) was assessed by asking the participant to position themselves parallel to the device being used for the Zoom call, lift the arm closest to the screen up in a straight line parallel to the sagittal plane, and stop at the furthest point without arching their back. A screenshot was taken at the furthest ROM and analyzed after the assessment with a goniometer. Each shoulder was measured twice, and the average score of each side was recorded.

Lower extremity flexibility

Hamstring flexibility was assessed through a seated one-leg extension and reach test with a yardstick or tape measure. The participant was given instructions to hold a hamstring stretch for 30 seconds on each side to familiarize them with the test and reduce the risk of injury. For the test, the protocol outlined by Jones et al. (1998) was followed. The only deviation from Jones et al.'s protocol was that the participant or a family member measured the distance to or from the toes while being instructed and observed by the assessor. The

participant had two attempts, and the highest attempt was recorded to the nearest 0.5 cm. The criterion validity of this test compared to the reference test (goniometer measurement of a passive straight leg raise) was 0.76 and 0.81 for males and females, respectively. The intraclass test-retest reliability was excellent, with 0.92 and 0.96 for males and females, respectively (Jones et al., 1998).

Balance

Balance was assessed through an eyes-open one-legged stand with a cap at 45 seconds that is validated and reliable(Franchignoni et al., 1998). For the test the protocol outlined by Franchignoni et al. (1998) was followed. Each leg was assessed once, and the time was recorded to the nearest 0.1 second (Springer et al., 2007).

Muscular Endurance

Muscular endurance was measured through the validated, and reliable 30-seconds sit-to-stand test (Eden et al., 2018; Jones et al., 1999). The test measures the times a subject can get up from a seated chair position in 30 seconds. The protocol outlined by Jones et al. was followed (Jones et al., 1999).

Cardiovascular Fitness

Cardiovascular fitness was assessed through the 2-minute step test. In this test the number of steps with the knee above the midway point from the patella to iliac crest in 2 minutes was counted. The protocol outlined by Rikli and Jones (1999) was followed. The participant was asked to complete the test perpendicular to the camera and ideally next to a background that allowed for easy detection of each leg during the Zoom call. This measure is commonly used in older adults and has a moderate validity to assess cardiovascular fitness (Johnston, 1999).

Patient Reported Outcomes

Patient-reported outcomes were administered at baseline and post-intervention through a closed survey on SurveyMonkey. Participants received an email with a unique survey link. The survey consisted of 78 questions, was optional, and did not include any incentives for completion. Participants' responses were de-identified by replacing their registration email addresses with study IDs for analysis. The description of the surveys used was based on the Checklist for Reporting Results of Internet E-Surveys (CHERRIES; Eysenbach, 2004).

Quality of Life (QOL)

QOL was assessed through the functional assessment of cancer therapy general scale (FACT-G). The change in the overall FACT-G score as well as the score of each domain subscale (physical well-being, social/family well-being, emotional well-being, and functional well-being) was evaluated. This tool has shown to be simple and fast to complete while still having high reliability and validity across cancer age groups (Cella et al., 1993; Overcash et al., 2001).

Fatigue

Fatigue was assessed through the functional assessment of chronic illness therapy fatigue scale (FACIT-F). The 13-item fatigue subscale has been commonly used in the cancer population and is reliable and valid (Yellen et al., 1997).

Barrier Self-Efficacy

Barrier self-efficacy was assessed using a scale developed based on the most common barriers for individuals living with and beyond cancer (Rogers et al., 2006). The scale consists of nine barriers, and the participant was asked how confident they are to exercise despite these barriers. The scale has a high internal consistency and good test-retest reliability (Rogers et al., 2006).

COVID-19 Impact

Stress, loneliness, and social support were assessed through the Perceived Stress Scale (PSS), the short form of the UCLA Loneliness Scale (ULS-6), and the Oslo Social Support Scale (OSSS-3), respectively. The 10-item PSS is a simple and widely established scale that has acceptable psychometric properties (Lee, 2012). The 6-item ULS-6 is a brief, reliable, and valid measure of loneliness (Neto, 2014). The 3-item OSSS-3 is a short, reliable and valid measure of social support (Kocalevent et al., 2018).

EXPLORATORY ANALYSIS

All data were analyzed using SPSS statistics (v26, IBM). For continuous data (age, BMI, MVPA, TMST, and change scores), normality was assessed by inspecting histograms, box plots, QQ-plots, the Shapiro-Wilk test of normality. Descriptive statistics are reported as mean and standard deviation (SD) for normally distributed data and medians and interquartile ranges (IQR) for non-normal distributed data. Categorical outcomes are reported as frequency and percentage. For pre-post intervention data, mean change scores were presented with 95% confidence intervals (95% CI) (Lee et al., 2014). Where available, change scores were compared with minimal clinically importance differences (MCIDs); the smallest difference that is perceived by patients as beneficial or harmful (Revicki et al., 2008). The proportion of participants improving or deteriorating by more than the MCID is also reported. Effect sizes (standardized mean difference) were calculated (Cohen's *d* or Hedge's *g*) and interpreted as 0.2 is small, 0.5 is moderate and 0.8 is large, in line with Cohen (1992).

References

Amireault, S., Godin, G., Lacombe, J., & Sabiston, C. M. (2015, Aug 12). The use of the Godin-Shephard Leisure-Time Physical Activity Questionnaire in oncology research: a systematic review. *Bmc Medical Research Methodology, 15*(1), 60. https://doi.org/ARTN 60
10.1186/s12874-015-0045-7

Campbell, K. L., Winters-Stone, K. M., Wiskemann, J., May, A. M., Schwartz, A. L., Courneya, K. S., Zucker, D. S., Matthews, C. E., Ligibel, J. A., & Gerber, L. H. (2019). Exercise guidelines for cancer survivors: consensus statement from international multidisciplinary roundtable. *Medicine & Science in Sports & Exercise, 51*(11), 2375-2390.

Cella, D. F., Tulsky, D. S., Gray, G., Sarafian, B., Linn, E., Bonomi, A., Silberman, M., Yellen, S. B., Winicour, P., & Brannon, J. (1993). The Functional Assessment of Cancer Therapy scale: development and validation of the general measure. *J Clin Oncol, 11*(3), 570-579.

Eden, M. M., Tompkins, J., & Verheijde, J. L. (2018). Reliability and a correlational analysis of the 6MWT, ten-meter walk test, thirty second sit to stand, and the linear analog scale of function in patients with head and neck cancer. *Physiotherapy theory and practice, 34*(3), 202-211.

Eysenbach, G. (2004). Improving the quality of Web surveys: the Checklist for Reporting Results of Internet E-Surveys (CHERRIES). *Journal of medical Internet research, 6*(3), e34.

Franchignoni, F., Tesio, L., Martino, M., & Ricupero, C. (1998). Reliability of four simple, quantitative tests of balance and mobility in healthy elderly females. *Aging Clinical and Experimental Research, 10*(1), 26-31.

Gell, N. M., Grover, K. W., Humble, M., Sexton, M., & Dittus, K. (2017). Efficacy, feasibility, and acceptability of a novel technology-based intervention to support physical activity in cancer survivors. *Supportive Care in Cancer, 25*(4), 1291-1300.

Hartman, S. J., Nelson, S. H., & Weiner, L. S. (2018). Patterns of Fitbit use and activity levels throughout a physical activity intervention: exploratory analysis from a randomized controlled trial. *JMIR mHealth and uHealth, 6*(2), e29.

Johnston, J. (1999). *Validation of a 2-minute step-in-place test relative to treadmill performance in older adults* [Unpublished master's thesis]. California State University.

Jones, C. J., Rikli, R. E., & Beam, W. C. (1999). A 30-s chair-stand test as a measure of lower body strength in community-residing older adults. *Research quarterly for exercise and sport, 70*(2), 113-119.

Jones, C. J., Rikli, R. E., Max, J., & Noffal, G. (1998). The reliability and validity of a chair sit-and-reach test as a measure of hamstring flexibility in older adults. *Research quarterly for exercise and sport, 69*(4), 338-343.

Kocalevent, R.-D., Berg, L., Beutel, M. E., Hinz, A., Zenger, M., Härter, M., Nater, U., & Brähler, E. (2018). Social support in the general population: standardization of the Oslo social support scale (OSSS-3). *BMC psychology, 6*(1), 31.

Lee, E.-H. (2012). Review of the psychometric evidence of the perceived stress scale. *Asian nursing research, 6*(4), 121-127.

Lee, E. C., Whitehead, A. L., Jacques, R. M., & Julious, S. A. (2014). The statistical interpretation of pilot trials: should significance thresholds be reconsidered? *Bmc Medical Research Methodology, 14*(1), 1-8.

Matthews, C. E., Chen, K. Y., Freedson, P. S., Buchowski, M. S., Beech, B. M., Pate, R. R., & Troiano, R. P. (2008). Amount of time spent in sedentary behaviors in the United States, 2003–2004. *American journal of epidemiology, 167*(7), 875-881.

Neto, F. (2014). Psychometric analysis of the short-form UCLA Loneliness Scale (ULS-6) in older adults. *European journal of ageing, 11*(4), 313-319.

Overcash, J., Extermann, M., Parr, J., Perry, J., & Balducci, L. (2001). Validity and reliability of the FACT-G scale for use in the older person with cancer. *American Journal of Clinical Oncology, 24*(6), 591-596.

Revicki, D., Hays, R. D., Cella, D., & Sloan, J. (2008). Recommended methods for determining responsiveness and minimally important differences for patient-reported outcomes. *Journal of clinical epidemiology, 61*(2), 102-109.

Rikli, R. E., & Jones, C. J. (1999). Development and Validation of a Functional Fitness Test for Community-Residing Older Adults. *Journal of Aging & Physical Activity, 7*(2), 129. https://ezproxy.lib.ucalgary.ca/login?url=https://search.ebscohost.com/login.aspx?direct=true&db=s3h&AN=6156314&site=ehost-live

Rogers, L. Q., Courneya, K. S., Verhulst, S., Markwell, S., Lanzotti, V., & Shah, P. (2006). Exercise barrier and task self-efficacy in breast cancer patients during treatment. *Supportive Care in Cancer, 14*(1), 84-90.

Springer, B. A., Marin, R., Cyhan, T., Roberts, H., & Gill, N. W. (2007). Normative values for the unipedal stance test with eyes open and closed. *Journal of geriatric physical therapy, 30*(1), 8-15.

Yellen, S. B., Cella, D. F., Webster, K., Blendowski, C., & Kaplan, E. (1997). Measuring fatigue and other anemia-related symptoms with the Functional Assessment of Cancer Therapy (FACT) measurement system. *Journal of pain and symptom management, 13*(2), 63-74.
