# Supplementary material for: The online delivery of exercise oncology classes supported with health coaching: a parallel pilot randomized controlled trial
Source: Pilot Feasibility Stud. 2023 May 12;9:82. doi: 10.1186/s40814-023-01316-z (PMC10175911; doi:10.1186/s40814-023-01316-z)
Supplement: Supplementary file 6 — Additional file 6. OSF2: Exploratory Results. [file 40814_2023_1316_MOESM6_ESM.pdf]

OSF2: Exploratory Results

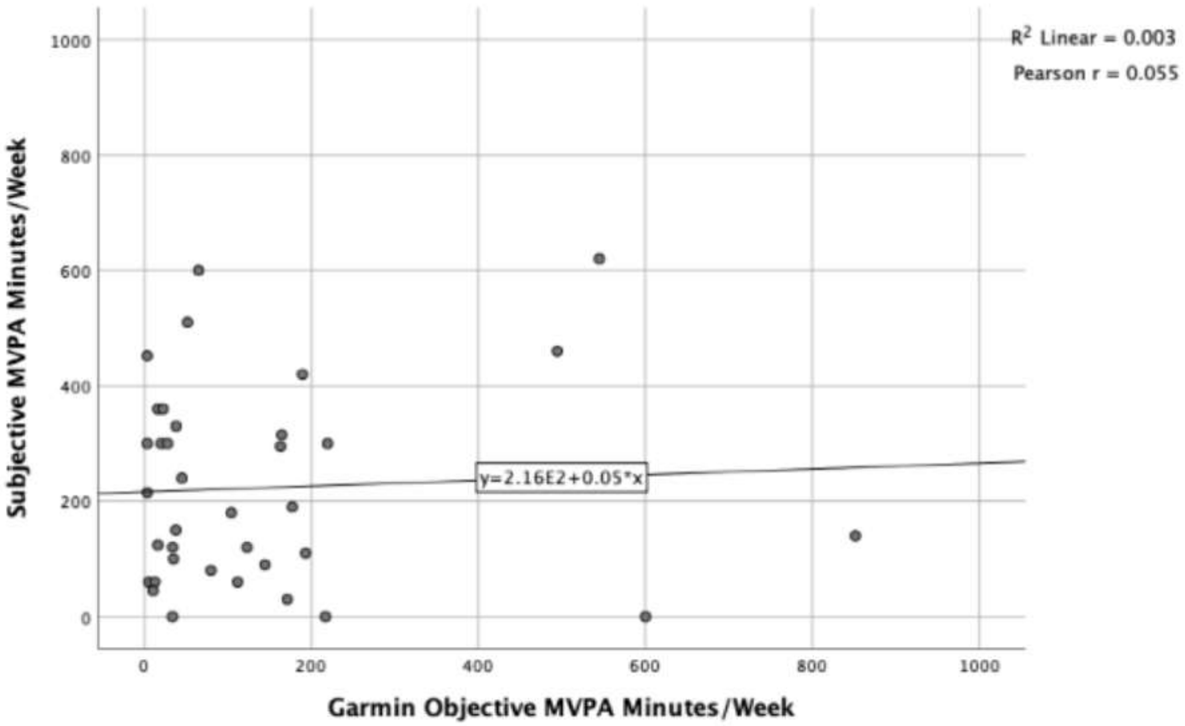

Figure 2. Correlation between the Subjective modified Godin Leisure Time Exercise Questionnaire (mGLTEQ) and the Objective Garmin Vivosmart4 to measure Moderate to Vigorous Physical Activity (MVPA) Minutes per Week

Table 1. Baseline to Post-intervention Differences for the non-HC Intervention Group in the 12-week Wave

	Baseline: Mean ± SD Median (IQR)	12WK Mean ± SD Median (IQR)	Mean ± SD / Median (IQR) Difference	95% CI	ES: *d*
FACT-G	71 ± 9	79 ± 8	8 ± 8	2.68 – 13.32	0.93
FACIT-F	36 ± 10	39 ± 6	3 ± 8	-3.20 – 8.70	0.33
Barrier SE	45 (37)	45 (22)	-1 ± 20	-21.55 – 21.79	0.00
OSSS-3	11 ± 2	11 ± 3	1 ± 2	-0.69 – 2.19	0.33
PSS	19 ± 4	15 ± 6	-4 ± 7	0.76 – 7.48	0.82
ULS-6	15 (5)	14 (8)	-2 (5)	-3.86 – 5.86	0.15
MVPA (min/week)	250.6 ± 166.1	219.4 ± 186.8	-31.3 ± 180.8	-79.23 – 141.73	0.18
BMI kg/m^2	26.6 (7.7)	26.5 (7.5)	-0.3 ± 1.1	-4.58 – 4.84	0.02
SROM (R.) (degree)	149.5 ± 12.0	151.0 ± 14.0	3.0 ± 14.5	-6.61 – 10.01	0.13
SROM (L.) (degree)	157.0 (7.0)	157.5 (7.5)	4.0 (6.5)	-3.96 – 4.96	0.07
Steps (reps)	115 ± 23	116 ± 20	-2.5 (28.5)	-11.91 – 14.91	0.07
Sit-to-Stand (reps)	15 ± 2	15 ± 2	0 ± 1.5	-1.25 – 1.85	0.13
Reach R. (cm)	2.5 (10.5)	9.5 (19.0)	3.5 (7.5)	-4.15 – 18.35	0.46
Reach L. (cm)	2.5 (13.0)	8.5 (21.0)	2.5 (6.5)	-6.31 – 18.11	0.34
Balance R (sec)	36.5 (30.2)	39.1 (27.4)	0.00 (4.7)	-10.57 – 25.37	0.26
Balance L (sec)	41.1 (28.3)	45.0 (28.1)	0.00 (18.5)	-13.57 – 21.37	0.14

ES=Effect size; Cohen's d of 0.2, 0.5, and 0.8 was judged as a small, moderate, and large effect size (Cohen, 1992)

FACT-G = Functional Assessment of Cancer Therapy – General (QoL); FACIT-F = Functional Assessment of Chronic Illness Therapy – Fatigue; Barrier SE = Barrier Self-Efficacy; OSSS-3 = Oslo Social Support Scale; PSS = Perceived Stress Scale; ULS-6 = UCLA Loneliness Scale; MVPA = Moderate to Vigorous Physical Activity; BMI = Body Mass Index; SROM = Shoulder Range of Motion; Reach = Sit and Reach test

Table 2. Baseline to Post-intervention Differences for the HC Intervention Group in the 12-week Wave

	Baseline: Mean ± SD Median (IQR)	12WK Mean ± SD Median (IQR)	Mean ± SD / Median (IQR) Difference	95% CI	ES: d / (g)
FACT-G	81 ± 7	81 ± 8	0 ± 6	-4.66 – 5.52	0.06
FACIT-F	41 ± 4	41 ± 4	0 ± 4	-2.77 – 3.05	0.03
BarrierSE	53 (23)	66 (33)	4 ± 28	-8.02 – 34.02	0.46
OSSS-3	11 ± 1	11 ± 2	0 ± 2	-0.95 – 1.23	0.09
PSS	13 ± 6	15 ± 4	2 ± 5	-1.71 – 5.71	0.40
ULS-6	15 (6)	16 (7)	-1 (1)	-3.54 – 5.54	0.15
MVPA (min/week)	165.0 ± 132.9	229.1 ± 150.8	64.1 ± 101	-34.27 – 162.55	0.45
BMI kg/m^2	24.5 (4.1)	25.0 (5.1)	-0.1 ± 0.6	-2.79 – 3.73	0.10
SROM (R.) (degree)	161.0 ± 10	157.5 ± 6.5	-1.5 ± 7.0	-3.16 – 9.76	0.39
SROM (L.) (degree)	151.0 (6.0)	162.5 (7.0)	-0.5 (18.5)	6.96 – 16.04	1.76
Steps (reps)	111 ± 9	119 ± 12	9 (9)	0.01 – 15.79	0.72
Sit-to-Stand (reps)	23 ± 7	24 ± 7	1 ± 5	-4.72 – 5.72	(0.07)
Reach R. (cm)	12.0 (8.5)	15.5 (13.0)	3.5 (5.0)	-4.84 – 11.84	0.32
Reach L. (cm)	9.5 (5.5)	14.5 (13.0)	3.0 (5.5)	-4.04 – 14.04	0.50
Balance R (sec)	45.0 (8.7)	45.0 (0.0)	0.0 (8.7)	-8.05 – 8.05	0.00
Balance L (sec)	45.0 (0.0)	45.0 (0.0)	0.0 (0.0)	0.00 – 0.00	0.00

ES=Effect size; Cohen's d of 0.2, 0.5, and 0.8 was judged as a small, moderate, and large effect size (Cohen, 1992)

FACT-G = Functional Assessment of Cancer Therapy – General (QoL); FACIT-F = Functional Assessment of Chronic Illness Therapy – Fatigue; Barrier SE = Barrier Self-Efficacy; OSSS-3 = Oslo Social Support Scale; PSS = Perceived Stress Scale; ULS-6 = UCLA Loneliness Scale; MVPA = Moderate to Vigorous Physical Activity; BMI = Body Mass Index; SROM = Shoulder Range of Motion; Reach = Sit and Reach test

Table 3. Baseline to Post-intervention Differences for the 12-Week Wave

Outcome Measure	Baseline (Mean ± SD Median (IQR))	12WK (Mean ± SD Median (IQR))	95% CI	ES: d or (g)
FACT-G	76 ± 9	80 ± 8	0.86 – 8.06	0.52
FACIT-F	40 (9)	40 (7)	-3.44 – 3.44	0.00
Barrier SE	52 ± 21	53 ± 19	-6.95 – 9.89	0.10
OSSS-3	11 (2)	11 (1)	-4.25 – 4.25	0.00
PSS	16 ± 6	15 ± 5	-0.92 – 3.44	0.24
ULS-6	15 ± 3	14 ± 4	-0.61 – 2.21	0.25
MVPA (min/week)	210.7 ± 152.7	223.9 ± 165.0	-52.22 – 78.74	0.08
BMI kg/m^2	26.7 ± 5.3	26.5 ± 5.2	-1.95 – 2.37	0.04
SROM (R.) (degree)	155.0 ± 12.5	154.0 ± 11.5	-4.21 – 5.55	0.06
SROM (L.) (degree)	156.0 (10.0)	158.5 (10.5)	-1.71 – 6.71	0.24
Steps (reps)	113 ± 17	118 ± 17	-2.47 – 11.39	0.26
Sit-to-Stand (reps)	16 (9)	18 (10)	-2.60 – 5.60	(0.16)
Reach R. (cm)	5.0 (12.0)	12.5 (15.0)	1.75 – 13.25	0.55
Reach L. (cm)	8.0 (12.0)	12.0 (19.5)	-3.53 – 13.59	0.25
Balance R (sec)	45.0 (17.1)	45.0 (7.7)	-7.02 – 7.02	0.00
Balance L (sec)	45.0 (12.1)	45.0 (0.0)	-6.70 – 6.70	0.00

ES=Effect size; Cohen's d / Hedge's g of 0.2, 0.5, and 0.8 was judged as a small, moderate, and large effect size (Cohen, 1992)
FACT-G = Functional Assessment of Cancer Therapy – General (QoL); FACIT-F = Functional Assessment of Chronic Illness Therapy – Fatigue; Barrier SE = Barrier Self-Efficacy; OSSS-3 = Oslo Social Support Scale; PSS = Perceived Stress Scale; ULS-6 = UCLA Loneliness Scale; MVPA = Moderate to Vigorous Physical Activity; BMI = Body Mass Index; SROM = Shoulder Range of Motion; Reach = Sit and Reach test

Table 4. Change Scores of the HC and Non-HC group for the 12-Week Wave

Outcome Measure	Non-HC: Mean ± SD Median (IQR)	HC: Mean ± SD Median (IQR)	95% CI	ES: g
FACT-G	8 ± 8	0 ± 6	1.51 - 13.63	1.04
FACIT-F	3 ± 8	0 ± 4	-4.53 – 10.31	0.44
Barrier SE	-1 ± 20	4 ± 28	-24.80 – 15.30	0.20
OSSS-3	1 ± 2	0 ± 2	-0.74 – 1.96	0.37
PSS	-4 ± 7	2 ± 5	-10.85 – -1.41	1.08
ULS-6	-2 (5)	-1 (1)	-4.87 – 3.87	0.13
MVPA (min/week)	-31.3 ± 180.8	64.1 ± 101	-218.03 – 27.25	0.64
BMI kg/m^2	-0.3 ± 1.1	-0.1 ± 0.6	-1.04 – 0.46	0.32
SROM (R.) (degree)	3.0 ± 14.5	-1.5 ± 7.0	-5.17 – 13.67	0.37
SROM (L.) (degree)	4.0 (6.5)	-0.5 (18.5)	-10.53 – 19.53	0.33
Steps (reps)	-3 (28)	9 (9)	-35.67 – 12.67	0.53
Sit-to-Stand (reps)	0 ± 2	1 ± 5	-3.53 – 4.53	0.15
Reach R. (cm)	3.5 (7.5)	3.5 (5.0)	-7.23 -7.23	0.00
Reach L. (cm)	2.5 (6.5)	3.0 (5.5)	-7.61 – 6.11	0.12
Balance R (sec)	0.0 (4.7)	0.0 (8.7)	-7.64 – 7.64	0.00
Balance L (sec)	0.0 (18.5)	0.0 (0.0)	-15.15 – 15.15	0.00

ES=Effect size; Cohen's d / Hedge's g of 0.2, 0.5, and 0.8 was judged as a small, moderate, and large effect size (Cohen, 1992)

FACT-G = Functional Assessment of Cancer Therapy – General (QoL); FACIT-F = Functional Assessment of Chronic Illness Therapy – Fatigue; Barrier SE = Barrier Self-Efficacy; OSSS-3 = Oslo Social Support Scale; PSS = Perceived Stress Scale; ULS-6 = UCLA Loneliness Scale; MVPA = Moderate to Vigorous Physical Activity; BMI = Body Mass Index; SROM = Shoulder Range of Motion; Reach = Sit and Reach test

Table 5. Percent of Participants Improving, Maintaining, or Worsening by the Minimal Clinically Important Difference for the 12-Week Wave

	Improved		Maintained		Worsen	
	HC *n* (%)	Non-HC *n* (%)	HC *n* (%)	Non-HC *n* (%)	HC *n* (%)	Non-HC *n* (%)
FACT-G	2 (28.6)	6 (75.0)	3 (42.9)	1 (12.5)	2 (28.6)	1 (12.5)
FACIT-F	2 (28.6)	3 (37.5)	4 (57.1)	3 (37.5)	1 (14.3)	2 (25.0)
Balance(R)	0 (0)	0 (0)	7 (100.0)	8 (100.0)	0 (0)	0 (0)
Balance(L)	0 (0)	1 (12.5)	7 (100.0)	7 (87.5)	0 (0)	0 (0)
SROM (R)	0 (0)	0 (0)	7 (100.0)	8 (100.0)	0 (0)	0 (0)
SROM (L)	1 (14.3)	1 (12.5)	6 (85.7)	7 (87.5)	0 (0)	0 (0)
MVPA	5 (71.4)	3 (37.5)	0 (0)	1 (12.5)	2 (28.6)	4 (50.0)
Sit-to-Stand	1 (16.7)	1 (12.5)	4 (66.7)	7 (87.5)	1 (16.7)	0 (0)
PSS	2 (28.6)	4 (50.0)	2 (28.6)	3 (37.5)	3 (42.9)	1 (12.5)

MCIDs: FACT-G = 3 points; FACIT-F = 3 points (McNeely et al., 2020); Balance = 24 seconds (Goldberg et al., 2011); SROM = >10°; MVPA = 26min/week (Hur et al., 2019); Sit-to-Stand = 2.6 repetitions (Wright et al., 2011); PSS-10 = 2.66 points (Drachev et al., 2020)
FACT-G = Functional Assessment of Cancer Therapy – General (QoL); FACIT-F = Functional Assessment of Chronic Illness Therapy – Fatigue; PSS = Perceived Stress Scale; MVPA = Moderate to Vigorous Physical Activity; SROM = Shoulder Range of Motion;
*Missing Values: Sit-to-Stand = 1 (HC).

Table 6. Baseline to Post-intervention Differences for the non-HC Intervention Group in the 8-Week Wave

	Baseline: Mean ± SD Median (IQR)	8WK Mean ± SD Median (IQR)	Mean ± SD / Median (IQR) Difference	g	95% CI
FACT-G	78.3 ± 8.7	80.8 ± 7.3	1.17 ± 5.31	0.31	-4.18 – 9.18
FACIT-F	36.8 ± 5.2	35.6 ± 12.1	-1.08 ± 9.61	0.13	-8.80 – 6.40
Barrier SE	46.00 (26.00)	38.50 (35.00)	-5.50 (28.0)	0.24	-32.87 – 17.87
OSSS-3	11.1 ± 2.4	10.8 ± 2.3	-0.50 ± 1.62	0.13	-2.25 – 1.65
PSS	15.0 (10.0)	14.5 (11.5)	-1.08 ± 4.44	0.05	-9.40 – 8.40
ULS-6	13.2 ± 3.9	13.9 ± 2.8	1.08 ± 2.87	0.20	-2.13 – 3.53
MVPA (min/week)	30.0 (225.0)	120.0 (252.5)	30.00 (120.00)	0.38	-108.54 – 288.54
BMI kg/m^2	27.6 ± 5.6	27.3 ± 6.5	0.11 (0.54)	0.05	-5.55 – 4.95
SROM (R.) (degree)	152.7 ± 8.3	152.1 ± 9.4	-0.45 ± 4.12	0.07	-8.09 – 6.89
SROM (L.) (degree)	151.2 ± 7.4	148.9 ± 6.7	-1.82 ± 7.17	0.32	-8.32 – 3.72
Steps (reps)	89.0 (23.3)	98.0 (32.0)	9.30 ± 10.23	0.32	-15.12 – 33.12
Sit to Stand (reps)	17.7 ± 5.9	18.6 ± 7.4	0.40 ± 2.59	0.14	-4.88 – 6.68
Reach R. (cm)	7.0 ± 12.1	7.9 ± 8.6	0.50 (7.50)	0.08	-8.15 – 9.95
Reach L. (cm)	6.7 ± 11.1	7.8 ± 8.4	1.50 (13.00)	0.11	-7.37 – 9.57
Balance R (sec)	38.9 (38.8)	45.0 (30.6)	0.00 (6.10)	0.17	-23.90 – 36.10
Balance L (sec)	45.0 (24.2)	21.4 (34.8)	0.00 (22.93)	0.79	-49.40 – 2.20

ES=Effect size; Hedge's g of 0.2, 0.5, and 0.8 was judged as a small, moderate, and large effect size (Cohen, 1992)

FACT-G = Functional Assessment of Cancer Therapy – General (QoL); FACIT-F = Functional Assessment of Chronic Illness Therapy – Fatigue; Barrier SE = Barrier Self-Efficacy; OSSS-3 = Oslo Social Support Scale; PSS = Perceived Stress Scale; ULS-6 = UCLA Loneliness Scale; MVPA = Moderate to Vigorous Physical Activity; BMI = Body Mass Index; SROM = Shoulder Range of Motion; Reach = Sit and Reach test

Table 7. Baseline to Post-intervention Differences for the HC Intervention Group in the 8-Week Wave

	Baseline: Mean ± SD Median (IQR)	8WK Mean ± SD Median (IQR)	Mean ± SD / Median (IQR) Difference	ES: d	95% CI
FACT-G	79.9 ± 14.1	84.8 ± 12.3	4.92 ± 6.87	0.37	-1.40 – 11.20
FACIT-F	36.9 ± 9.5	40.9 ± 8.5	4.00 ± 6.93	0.44	-0.28 – 8.28
Barrier SE	38.00 (43.00)	61.00 (37.00)	5.50 (42.75)	0.57	3.84 – 42.16
OSSS-3	11.0 ± 2.3	10.9 ± 2.1	-0.08 ± 1.24	0.05	-0.94 – 1.14
PSS	17.0 (12.8)	13.5 (7.8)	-1.33 ± 4.74	0.33	-2.18 – 9.18
ULS-6	12.6 ± 4.0	13.3 ± 3.1	0.75 ± 1.71	0.20	-1.05 – 2.45
MVPA (min/week)	90.0 (172.5)	257.5 (309.0)	85.00 (221.25)	0.67	28.46- 306.54
BMI kg/m^2	27.4 ± 5.7	26.8 ± 5.4	-0.33 (0.89)	0.11	-2.02 – 3.22
SROM (R.) (degree)	157.0 ± 13.4	157.0 ± 10.3	0.00 ± 6.64	0.00	-5.87 – 5.87
SROM (L.) (degree)	158.6 ± 8.9	158.2 ± 7.5	-0.42 ± 6.99	0.05	-3.55 – 4.35
Steps (reps)	83.5 (16.5)	96.5 (18.8)	8.67 ± 12.24	0.73	4.58 – 21.42
Sit to Stand (reps)	16.8 ± 3.2	17.3 ± 4.8	0.42 ± 5.16	0.12	-1.61 – 2.61
Reach R. (cm)	6.9 ± 8.7	9.3 ± 9.1	4.25 (5.38)	0.27	-1.80 – 6.60
Reach L. (cm)	6.8 ± 7.5	9.7 ± 7.4	4.00 (6.38)	0.39	-0.61 – 6.41
Balance R (sec)	45.0 (18.8)	45.0 (16.5)	0.00 (5.25)	0.00	-8.42 – 8.42
Balance L (sec)	45.0 (0.0)	45.0 (25.0)	0.00 (3.15)	0.00	-15.88 – 15.88

ES=Effect size; Cohen's d of 0.2, 0.5, and 0.8 was judged as a small, moderate, and large effect size (Cohen, 1992)

FACT-G = Functional Assessment of Cancer Therapy – General (QoL); FACIT-F = Functional Assessment of Chronic Illness Therapy – Fatigue; Barrier SE = Barrier Self-Efficacy; OSSS-3 = Oslo Social Support Scale; PSS = Perceived Stress Scale; ULS-6 = UCLA Loneliness Scale; MVPA = Moderate to Vigorous Physical Activity; BMI = Body Mass Index; SROM = Shoulder Range of Motion; Reach = Sit and Reach test

Table 8. Baseline to Post-intervention Differences for the 8-Week Wave

Outcome Measures	8-Week Wave			
	Baseline (Mean ± SD Median (IQR))	8WK (Mean ± SD Median (IQR))	ES: g	95% CI
FACT-G	79.08 ± 11.87	82.79 ± 10.13	0.34	-2.64 – 10.06
FACIT-F	36.84 ± 7.39	38.25 ± 10.61	0.15	-3.83 – 6.65
Barrier SE	46.56 ± 23.06	48.88 ± 20.58	0.11	-10.26 – 14.90
OSSS-3	11.04 ± 2.32	10.83 ± 2.14	0.09	-1.49 – 1.07
PSS	15.44 ± 6.61	14.00 ± 6.26	0.22	-5.14 – 2.26
ULS-6	12.88 ± 3.88	13.63 ± 2.89	0.22	-1.22 – 2.72
MVPA (min/week)	90.00 (215.00)	185.00 (234.00)	0.42	-34.07 – 224.07
BMI kg/m^2	27.50 ± 5.54	27.01 ± 5.76	0.09	-3.81 – 2.83
SROM (R.) (degree)	154.74 ± 10.99	154.65 ± 9.97	0.01	-6.21 – 6.03
SROM (L.) (degree)	154.74 ± 8.80	153.71 ± 8.41	0.12	-6.04 – 3.98
Steps (reps)	88.46 ± 16.17	97.39 ± 19.42	0.50	-1.55 – 19.41
Sit to Stand (reps)	17.25 ± 4.69	17.87 ± 6.10	0.11	-2.57 – 3.81
Reach R. (cm)	6.94 ± 10.38	8.64 ± 8.67	0.18	-3.88 – 7.28
Reach L. (cm)	6.76 ± 9.39	8.76 ± 7.79	0.23	-3.04 – 7.04
Balance R (sec)	45.00 (30.00)	45.00 (22.00)	0.00	-15.40 – 15.40
Balance L (sec)	45.00 (16.00)	45.00 (32.8)	0.00	-15.27 – 15.27

ES=Effect size; Cohen's d / Hedge's g of 0.2, 0.5, and 0.8 was judged as a small, moderate, and large effect size (Cohen, 1992)

FACT-G = Functional Assessment of Cancer Therapy – General (QoL); FACIT-F = Functional Assessment of Chronic Illness Therapy – Fatigue; Barrier SE = Barrier Self-Efficacy; OSSS-3 = Oslo Social Support Scale; PSS = Perceived Stress Scale; ULS-6 = UCLA Loneliness Scale; MVPA = Moderate to Vigorous Physical Activity; BMI = Body Mass Index; SROM = Shoulder Range of Motion; Reach = Sit and Reach test

Table 9. Change Scores of the HC and Non-HC group for both the 8-Week Wave

Outcome Measures	8-Week Wave			
	Non-HC: Mean ± SD Median (IQR)	HC: Mean ± SD Median (IQR)	ES: d or (g)	95% CI
FACT-G	1.17 ± 5.31	4.92 ± 6.87	0.61	0.74 – 6.76
FACIT-F	-1.08 ± 9.61	4.00 ± 6.93	0.61	0.88 – 9.28
Barrier SE	-5.50 (28.0)	5.50 (42.75)	0.30	-7.79 – 29.79
OSSS-3	-0.50 ± 1.62	-0.08 ± 1.24	0.29	-0.29 – 1.13
PSS	-1.08 ± 4.44	-1.33 ± 4.74	0.05	-1.92 – 2.42
ULS-6	1.08 ± 2.87	0.75 ± 1.71	0.14	-0.95 – 1.61
MVPA (min/week)	30.00 (120.00)	85.00 (221.25)	0.31	-45.11 – 155.11
BMI kg/m^2	0.11 (0.54)	-0.33 (0.89)	(0.58)	-1.11 – 0.23
SROM (R.) (degree)	-0.45 ± 4.12	0.00 ± 6.64	(0.08)	-4.40 – 5.30
SROM (L.) (degree)	-1.82 ± 7.17	-0.42 ± 6.99	(0.20)	-4.74 – 7.54
Steps (reps)	9.30 ± 10.23	8.67 ± 12.24	(0.06)	-10.79 – 9.53
Sit to Stand (reps)	0.40 ± 2.59	0.42 ± 5.16	(0.00)	-3.73 – 3.77
Reach R. (cm)	0.50 (7.50)	4.25 (5.38)	(0.58)	-1.87 – 9.37
Reach L. (cm)	1.50 (13.00)	4.00 (6.38)	(0.25)	-6.26 – 11.26
Balance R (sec)	0.00 (6.10)	0.00 (5.25)	(0.00)	-4.92 – 4.92
Balance L (sec)	0.00 (22.93)	0.00 (3.15)	(0.00)	-13.90 – 13.90

ES=Effect size; Cohen's d / Hedge's g of 0.2, 0.5, and 0.8 was judged as a small, moderate, and large effect size (Cohen, 1992)

FACT-G = Functional Assessment of Cancer Therapy – General (QoL); FACIT-F = Functional Assessment of Chronic Illness Therapy – Fatigue; Barrier SE = Barrier Self-Efficacy; OSSS-3 = Oslo Social Support Scale; PSS = Perceived Stress Scale; ULS-6 = UCLA Loneliness Scale; MVPA = Moderate to Vigorous Physical Activity; BMI = Body Mass Index; SROM = Shoulder Range of Motion; Reach = Sit and Reach test

Table 10. Percent of Participants Improving, Maintaining, or Worsening by the Minimal Clinically Important Difference for the 8-Week Wave

	Improved		Maintained		Worsen	
	HC n (%)	Non-HC n (%)	HC n (%)	Non-HC n (%)	HC n (%)	Non-HC n (%)
FACT-G	6 (50.0)	6 (50.0)	4 (33.3)	3 (25.0)	2 (16.7)	3 (25.0)
FACIT-F	8 (66.7)	6 (50.0)	3 (25.0)	1 (8.3)	1 (8.3)	5 (41.7)
Balance(R)	1 (8.3)	0 (0.0)	11 (91.7)	11 (100.0)	0 (0.0)	0 (0.0)
Balance(L)	0 (0.0)	0 (0.0)	11 (91.7)	9 (81.8)	1 (8.3)	1 (9.1)
SROM (R)	0 (0.0)	0 (0.0)	12 (100.0)	11 (100.0)	0 (0.0)	0 (0.0)
SROM (L)	0 (0.0)	0 (0.0)	12 (100.0)	11 (100.0)	0 (0.0)	0 (0.0)
MVPA	9 (75.0)	8 (66.7)	2 (16.7)	2 (16.7)	1 (8.3)	2 (16.7)
SittoStand	3 (25.0)	2 (20.0)	7 (58.3)	7 (70.0)	2 (16.7)	1 (10.0)
PSS-10	4 (33.3)	5 (41.7)	6 (50.0)	4 (33.3)	2 (16.7)	3 (25.0)

MCIDs: FACT-G = 3 points; FACIT-F = 3 points (McNeely et al., 2020); Balance = 24 seconds (Goldberg, Casby, & Wasielewski, 2011); SROM = >10°; MVPA = 26min/week (Hur et al., 2019); Sit to Stand = 2.6 repetitions (Wright, Cook, Baxter, Dockerty, & Abbott, 2011); PSS-10 = 2.66 points (Drachev et al., 2020)

*Missing Values: FACT-G = 1 (Non-HC); FACIT-F = 1 (Non-HC); Balance (R) = 2 (Non-HC); Balance (L) = 2 (Non-HC); SROM (R) = 2 (Non-HC); SROM (L) = 2 (Non-HC); MVPA = 1 (Non-HC); Sit to Stand = 3 (Non-HC); PSS-10 = 1 (Non-HC).
